# NF-κB Plays a Key Role in Inducing *CD274* Expression in Human Monocytes after Lipopolysaccharide Treatment

**DOI:** 10.1371/journal.pone.0061602

**Published:** 2013-04-09

**Authors:** Gang Huang, Qianjun Wen, Yongliang Zhao, Qiangguo Gao, Yun Bai

**Affiliations:** 1 Department of Medical Genetics, Third Military Medical University, Chongqing, China; 2 Department of Infectious Diseases, Affiliated Hospital of Guiyang Medical College, Guiyang, Guizhou, China; 3 Department of General Surgery, Southwest Hospital, Chongqing, China; 4 Department of Cell Biology, Third Military Medical University, Chongqing, China; National Cancer Institute (INCA), Brazil

## Abstract

CD274, one of two co-stimulatory ligands for programmed death 1 and widely expressed in the mononuclear phagocyte system (MPS), may co-stimulate T cells and regulates inflammatory responses. However, changes in *CD274* gene expression and the underlying molecular mechanism are poorly understood during inflammatory responses. Therefore, delineation of the complex mechanisms regulating *CD274* expression is critical to understand this immunoregulatory system during inflammatory responses. The purpose of this study was to assess the molecular mechanisms regulating *CD274* expression in an *in vitro* monocyte model of inflammatory response. Firstly, *CD274* expression levels in human primary monocytes after lipopolysaccharide (LPS) treatment were observed and correlated with NF-κB activation. Secondly, based on the distribution of putative NF-κB binding sites, 5′ truncated human *CD274* promoter reporters were constructed, transfected into U937 cells and critical promoter regions for basal (nt −570 to +94) and LPS-induced (nt −1735 to −570) transcription were identified by dual luciferase assays. Finally, a key NF-κB binding site (nt −610 to −601) for LPS-inducible *CD274* transcriptional activity was characterized by point mutation analysis and chromatin immunoprecipitation analysis assays (ChIP). Thus, the present study establishes a molecular basis to understand the mechanisms governing *CD274* expression in certain infections and inflammatory disorders.

## Introduction

The primary inflammatory response induced by gram-negative bacteria involves activation of the innate immune system. This activation can then trigger systemic inflammatory response syndrome (SIRS) by releasing a cascade of proinflammatory cytokines, thereby causing high morbidity and mortality [1], [2]. Recently, mounting evidence suggests that dysfunction of the adaptive immune system is also involved in SIRS, which is characterized by T cell anergy [1], [3]. As an important class of antigen presenting cells for T cells and the major component of the innate immune system, the mononuclear phagocyte system (MPS) regulates the inflammatory response and immune functions via membrane proteins and secreted cytokines such as B7, TNF-α (tumor necrosis factor-alpha), IL-6 (interleukin-6) and IL-10 (interleukin-10) [4]. *CD274*, an important member of the B7 superfamily, is expressed in the MPS and plays a key role in the pathogenesis of inflammatory diseases [5], [6]. After binding to its cognate receptor, *CD274* exerts inflammation regulatory functions via a negative co-stimulatory effect on T cell functions to inhibit cytokine secretion, facilitate apoptosis of activated T cells and induce T cell anergy [7], [8].

Lipopolysaccharide (LPS), the main cell wall component of gram-negative bacteria, strongly induces the production and release of various cytokines and inflammatory factors that initiate the inflammation process. Recently, Yamazaki and colleagues found that LPS up-regulates *CD274* gene expression in the mouse MPS [9]. However, the mechanisms underlying this effect are still unknown. Further exploration of its molecular mechanism is necessary to better understand the pathogenesis of inflammation. It has been widely reported that NF-κB (nuclear factor kB) activation is involved in numerous inflammatory processes, and that NF-κB is a key molecule in the LPS-TLR4 (Toll-like receptor 4) signal transduction pathway [10], [11]. Based on *in silico* analysis, the putative promoter region of the human *CD274* gene contains four potential NF-κB binding sites, indicating that NF-κB may play an essential role in LPS-induced *CD274* gene expression in the MPS.

In the present study, we show evidence indicating that NF-κB binds to the human *CD274* promoter and regulates its transcription in human monocytes after LPS treatment, and this regulation is most likely mediated via one of the NF-κB binding sites (nt −610 to −601). Our study establishes a molecular basis to understand the mechanisms governing *CD274* expression in certain infections and inflammation disorders.

## Materials and Methods

### Cells, Culture Conditions and Treatments

Primary human monocytes were obtained from healthy donors with written informed consent, and this study was approved by the Medical Ethics Committees of Third Military Medical University plus conducted in accordance with the ethical guidelines of the Declaration of Helsinki. Fresh whole blood was drawn into vacutainer tubes (Becton Dickinson & Co., Franklin Lakes, NJ, USA) containing EDTA. Peripheral blood mononuclear cells (PBMCs) were isolated using Ficoll-Hypaque (TBD, China). CD14+ monocytes were isolated from PBMCs by negative selection using a Human Monocyte Isolation Kit II (Miltenyi, Germany) according to the manufacturer's instructions. Monocyte purity was verified as >90% by anti-CD14 staining (Cat. #11-0149; eBioscience, San Diego, CA, USA) using flow cytometry. Cells were seeded at 1×10^6^ cells/well in 12 well plates (Corning, NY, USA), maintained in RPMI 1640 medium (Gibco BRL, Grand Island, NY, USA) supplemented with 10% fetal calf serum (Hyclone, Logan, UT, USA), and activated by 400 ng/ml LPS (*Escherichia coli* serotype O111:B4; Sigma, Batavia, IL, USA). Cells were harvested at 0, 2, 6, 12 and 24 h. For dual luciferase assays, the human monocytic cell line U937 (CRL-1593; ATCC, Manassas, VA, USA) was used. U937 cells were cultured as described for primary human monocytes.

### Inhibition of the LPS-TLR4 Signaling Sub-pathway in Primary Human Monocytes

Primary human monocytes were pre-treated with several known inhibitors of the LPS-TLR4 signaling pathway. NF-κB inhibitor BAY (BAY 11-7082; Sigma-Aldrich, St. Louis, MO, USA), AP-1 inhibitor T-5224 (Toyama Chemical, Japan) and PI3K inhibitor Wort (Wortmannin; Alexis, San Diego, CA, USA) were used to assess their effects on individual signal protein activation and *CD274* mRNA accumulation after LPS treatment. All inhibitors were dissolved in dimethylsulfoxide (DMSO). Monocytes were pre-treated with BAY (20 µM), T-5224 (20 µM), Wort (30 µM) or vehicle only (0.1% DMSO) individually for 50 min and then incubated with 400 ng/ml LPS for 12 h followed by *CD274* mRNA quantification, or incubated with 400 ng/ml LPS for 24 h followed by CD274 protein immunobloting assays. The final concentration of DMSO in cell culture medium was 0.1%.

### Transfection of Primary Human Monocytes with Small Interfering RNA (siRNA) against p65

NF-κB was inhibited further with an siRNA against the p65. siRNA against the human *p65* gene (Cat. #6261) and an RNAi-negative control (Cat. #6201) were purchased from Cell Signaling, USA. Given that NF-κB signaling is required for cell survival, we should firstly find an appropriate concentration of p65 siRNA to nucleofect the monocytes, in order to on the one hand effectively knockdown the p65 gene expression and on the other hand, have modest impact on the cell viability. A series of concentrations of siRNA (0, 20, 40, 60, 80, 100 nM) were nucleofected separately into primary human monocytes with a Lonza apparatus as described elsewhere [12], [13], then the cells were incubated for 48 h, gene silencing effect was assayed by western blotting, and the cells' viability was evaluated by CCK-8 (Cell Counting Kit-8; Dojindo, Japan) according to the manufacturer's instructions. After the appropriate concentration of p65 siRNA (40–60 nM) was determined (Fig. S1), siRNAs (50 nM) was nucleofected into primary human monocytes, then LPS (400 ng/ml) or control medium was added after 24 h of transfection, then incubated for 0, 12, 24 and 48 h. Gene silencing was confirmed by western blotting again.

### *CD274* mRNA Quantification by Real-Time PCR

Total RNA from primary monocytes was isolated with TRIzol reagent (1×10^6^ cells/ml; Invitrogen, Grand Island, NY, USA). First strand cDNA was synthesized by extension of an oligo(dT)_18_ primer with 200 U Superscript III (Invitrogen, USA) in a mixture containing 1 µg total RNA. Primer sequences are shown in Table 1. All primers were evaluated by conventional PCR and shown to amplify only one product, as visualized in 2% agarose gels. Ten nanograms of template cDNA and 100 nM each primer was used for PCR. Real-time quantitative PCR was performed on an ABI 7500 Fast Real-Time PCR System (Applied Biosystems, USA) using SYBR Green PCR Master Mix (TOYOBO, Japan) with detection according to the manufacturer's instructions. Quantitative PCR consisted of an initial cycle of 95°C for 10 min, and then 40 cycles of 95°C for 20 s and 68°C for 30 s. Gene expression was normalized to the housekeeping gene, glyceraldehyde-3-phosphate dehydrogenase (GAPDH). Results were represented as fold changes (arbitrary units) relative to a control group.

**Table 1:** Primer sequences for constructs, RACE, real-time PCR, ChIP PCR.

Designation	Primers/Probes Sequence	Amplicon Size (bp)
CD274-qPCR	(F)5′-GGTGGTGCCGACTACAAGCGA-3′ (R)5′-CCTTGGGGTAGCCCTCAGCCT-3′	133
GAPDH-qPCR	(F)5′-GGGGAAGGTGAAGGTCGGAGT-3′ (R)5′-TCTCGCTCCTGGAAGATGGTGAT-3	240
CD274-5′RACE	(F) provided in the Smart RACE kit (R)5′-CTGTGATCTGAAGTGCAGCATTTC-3′	
pGL3-2097Luc	(F) 5′-GGGGTACC^a^ ACTGCTCTTCTCCCATCTCA-3′ (R)5′-CCGCTCGAG b AAGCTGCGCAGAACT-3′	2, 165
pGL3-1735Luc	(F) 5′-GGGGTACC^a^ GTAGACCCTGAACACTGCT-3′ (R)5′-CCGCTCGAG^b^ AAGCTGCGCAGAACT-3′	1, 805
pGL3-1277Luc	(F) 5′-GGGGTACC^a^ TTCGGGAACTTTGGGAAG-3′ (R)5′-CCGCTCGAG^b^ AAGCTGCGCAGAACT-3′	1, 346
pGL3-570Luc	(F) 5′-GGGGTACC^a^ TATGTCGAGGAACTTTGAGGA-3′ (R)5′-CCGCTCGAG^b^ AAGCTGCGCAGAACT-3′	640
pGL3-91Luc	(F) 5′-GGGGTACC^a^ GATTTCACCGAAGGTCAGG-3′ (R)5′-CCGCTCGAG^b^ AAGCTGCGCAGAACT-3′	160
pGL3-51Luc	(F) 5′-GGGGTACC^a^ TGGATTTGCTGCCTTG-3′ (R)5′-CCGCTCGAG^b^ AAGCTGCGCAGAACT-3′	120
pGL3-1735Luc(M1)	(F) 5′-CACAGTCACCAAAGTTCTCTT^c^ GTCACCCAACTTCGG-3′ (R)5′-CCGAAGTTGGGTGACAAGAG^c^ AACTTTGGTGACTGTG-3′	6, 610
pGL3-1735Luc(M2)	(F) 5′-AGATGTAGCTCGGGATCTCTT^c^ GTTCTTTTAATGACA-3′ (R)5′-TGTCATTAAAAGAACAAGAG^c^ ATCCCGAGCTACATCT-3′	6, 610
pGL3-1735Luc(M3)	(F) 5′-GATTTCACCGAAGGTCACTCTC^c^ ACGCCCGGCAAACTG-3′ (R)5′-CAGTTTGCCGGGCGTGAGAG^c^ TGACCTTCGGTGAAATC-3′	6, 610
ChIP(site−610 to−601)	(F)5′-CTTCCGCAGCCTTAATCCTTA-3′ (R)5′-ATCGTGGATTCTGTGACTTCCTC-3′	151(−684/−534#)

# Positions given are relative to *CD274* transcription start site.

a Underlined nucleotides represent KpnI site.

b Underlined nucleotides represent XhoI site.

c Bold-type nucleotides represent introduced desired mutations of NF-κB binding site.

### Western Blot Assay for Detection of Protein Expression and Nuclear Translocation

To study the time kinetics of NF-κB activation, primary human monocytes were harvested at 0, 0.5, 1, 1.5 and 2 h after LPS treatment. Nuclear and cytoplasmic proteins were extracted by a Nuclear-Cytosol Extraction Kit (Applygen Technologies, China) for western blot analysis. Protein concentration was determined using BCA reagents (Pierce, USA) according to the manufacturer's instructions. Thirty micrograms of cytoplasmic protein extract and 20 µg nuclear protein extract were denatured in Laemmli buffer and separated using 12% SDS-polyacrylamide gel electrophoresis. Then, proteins were transferred in a transfer buffer for 1 h using a Bio-Rad Semi-Dry apparatus. Washes and incubations were performed according to standard procedures. Anti-CD274 monoclonal antibody (Clone MIH1, 1∶1000) and anti-human NF-κB p65 polyclonal antibody (1∶1500) were purchased from eBioscience, USA. Anti-IκB-α monoclonal antibody (sc-56710, 1∶1000) was purchased from Santa Cruz, USA. Anti-TATA binding protein (TBP) antibody (ab52701, 1∶1000) was purchased from Abcam, UK. Horseradish peroxidase (HRP)-labeled anti-GAPDH monoclonal antibody (Clone KC-5G5, 1∶5000) was purchased from KangChen Bio-tech, China, and HRP-labeled anti-mouse IgG (1∶3000) was purchased from Zhongshan Bio-tech, China. Western blots were visualized by ECL reagent (Pierce Biotechnology, USA) in a Storm 860 PhosphorImager.

### Flow Cytometric Assay of CD274 Protein Expression

Cell surface expression of CD274 on human primary monocytes was quantified by flow cytometry on a FACSCalibur (BD, Germany) using CellQuest® software. A total of 10,000 events were evaluated. Histograms were analyzed by calculating the geometric mean fluorescence (geometric mean specific stain/geometric mean isotype control). Phycoerythrin (PE) anti-human CD274 monoclonal antibody (Cat. #12-5983) and PE mouse IgG1 Isotype Control (Cat. #12-4714) were purchased from eBioscience, USA.

### Rapid amplification of cDNA ends (RACE)

The 5′-end of *CD274* was obtained using a SMART RACE cDNA Amplification Kit (Clontech, USA) according to the manufacturer's instructions. Briefly, the reverse transcription reaction was performed using 1 µg total RNA prepared from primary human monocytes by incubation at 42°C for 1.5 h and stopped by heating to 72°C for 7 min. PCR was performed for 35 cycles (conditions: 94°C×30 s, 68°C×30 s, 72°C×3 min). The 5′ consensus primer was provided in the kit. The *CD274* coding region primer used for 3′ priming was: 5′-CTGTGATCTGAAGTGCAGCATTTC-3′. Appropriately sized PCR product bands were subcloned into a pGEM-T Easy Vector (Promega, USA) and ten clones were sequenced. Sequencing results were compared with that of the human genome sequence using the BLAST algorithm, and the corresponding first base of the longest clone was considered as the transcription start site (TSS) of *CD274*.

### Cloning and *in silico* Sequence Analysis of a Putative *CD274* Promoter Region

An approximately 2.1 kb fragment upstream of the *CD274* TSS was amplified by PCR using normal human genomic DNA as a template and KOD-plus DNA polymerase according to the manufacturer's instructions (TOYOBO, Japan). The forward primer included a KpnI site and two protecting bases, and the reverse primer included a XhoI site and three protecting bases as shown in Table 1. PCR was performed for 35 cycles (conditions: 98°C×20 s, 60°C×30 s, 68°C×2 min). PCR products were digested by KpnI and XhoI and then directionally subcloned into a pGL3-Basic luciferase reporter vector (Promega, USA). The construct was confirmed by sequencing and denoted as pGL3-2097Luc. Potential binding sites for transcription factor NF-κB were predicted with the CONSITE program (http://asp.ii.uib.no:8090/cgi-bin/CONSITE/consite), which searches highly correlated sequence fragments against the profiling data of TRANSFAC resources [14].

### Deletion Mutant Plasmid Constructs

PCR was employed to produce serial deletions in the 5′ region of pGL3-2097Luc according to the distribution of potential NF-κB binding sites. Primers are shown in Table 1. PCR conditions were the same as those for cloning the 2097Luc fragment. Amplicons were then ligated into KpnI/XhoI sites of the pGL3-Basic plasmid in the forward orientation, upstream of a luciferase reporter to generate pGL3-1735Luc, pGL3-1277Luc, pGL3-570Luc, pGL3-91Luc and pGL3-51Luc. All constructs were confirmed by DNA sequencing.

### Transient Transfection and Dual Luciferase Assay

U937 cells were plated (10^6^ cells/well) in 6 well plates (Corning, USA) 24 h prior to transfection. Serial *CD274* promoter constructs and pGL3-Basic vector (2 µg DNA/each) as the control were transiently cotransfected with 0.2 µg pRL-TK using a Lonza apparatus according to the manufacturer's instructions. At 24 h post-transfection, cells were treated with 400 ng/ml LPS for 24 h, then firefly and renilla luciferase activities were measured with a Luminoskan Ascent luminometer (Thermo Labsystems, Finland). Luminescence experiments were performed at least three times, with each transfection performed in triplicate using seven separate DNA preparations. Results were expressed as fold increases in the ratio of luciferase activity (RLA) of the *CD274* promoter construct vectors compared with that of the RLA of pGL3-Basic.

### Mutation Analysis of the pGL3-1735Luc Promoter

Primers containing mutant NF-κB binding sites 1 (M1), 2 (M2) or 3 (M3) were designed to create mutant pGL3-1735Luc constructs as shown in Table 1. Mutagenesis of wild-type pGL3-1735Luc was performed using a QuikChange site-directed mutagenesis kit (Stratagene, USA). The integrity of each vector and the presence of desired mutations were verified by DNA sequencing. These mutant vectors were referred to as pGL3-1735Luc(M1), pGL3-1735Luc(M2) and pGL3-1735Luc(M3). The mutant reporter gene constructs were transiently cotransfected with pRL-TK into U937 cells using a Lonza apparatus, then treated with 400 ng/ml LPS and analyzed as described above. Wild-type pGL3-1735Luc was used as a control reporter plasmid.

### Chromatin Immunoprecipitation (ChIP) Assay

ChIP assays were performed using a ChIP Assay kit (Millipore, USA) according to the manufacturer's instructions. Briefly, approximately 1×10^7^ human primary monocytes were first treated with or without 400 ng/ml LPS for 1 h and then fixed with formaldehyde. After washing, cells were lysed in 200 µl SDS lysis buffer, and lysates were subjected to sonication on ice to shear DNA to lengths between 400 and 600 bp. Sonicated DNA was diluted 10-fold in ChIP dilution buffer. A fraction of the diluted supernatant (∼20 µl) was transferred to a new tube and kept as a template for PCR. Protein/DNA complexes were recovered by phenol/chloroform extraction. NF-κB/DNA complexes were pulled down using an anti-NF-κB p65 antibody (Cat. #06-418, Millipore, USA). Anti-β-actin (Cat. sc-47778X, Santa Cruz, USA), anti-STAT1 (signal transducer and activator of transcription 1) (Cat. # 9172S, Cell Signaling Technology, USA) and IgG antibodies (Cat. # ab37415, Abcam, UK) were used to demonstrate non-specific precipitation (negative control). The resultant precipitates were then used as templates to amplify the *CD274* promoter sequence containing the NF-κB binding motif (site −610 to −601). Primers for ChIP PCR are shown in Table 1. Real-time quantitative PCR was performed as previously mentioned to measure the relative enrichment efficiency of the different amplicons in the total input ChIP DNA fragments. Results were represented as fold changes (arbitrary units) relative to the input group.

### Statistical Analysis

Data were represented as the means ± SEM of at least three independent experiments. The significance of differences between independent means was assessed by the Student's unpaired t-test (two-tailed). Analysis of variance was performed on results from more than two groups. A value of p<0.05 was considered statistically significant.

## Results

### NF-κB Plays a Key Role in the Up-regulation of *CD274* Expression in Primary Human Monocytes after LPS Treatment

**Figure 1:**
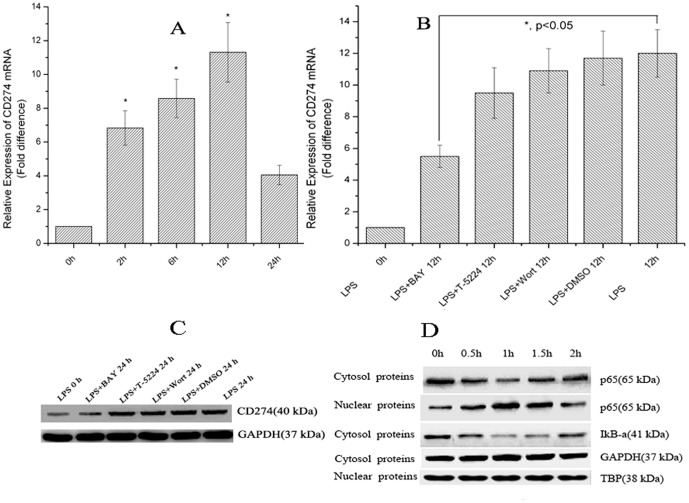
LPS treatment regulates CD274 mRNA levels mainly via the NF-κB signaling pathway in primary human monocytes. CD274 mRNA levels were determined by quantitative real-time PCR and are shown as fold changes in arbitrary units. (A) LPS treatment regulates CD274 mRNA levels (n = 3, repeated three times, *P<0.05 vs. control without LPS treatment). (B) Inhibitory effects of BAY (an NF-κB inhibitor), T-5224 (an AP-1 inhibitor), Wort (a PI3K inhibitor) and DMSO on CD274 mRNA levels after LPS treatment (n = 3, repeated three times, *P<0.05 vs. control with LPS treatment for 12 h). (C) Inhibitory effects of BAY, T-5224, Wort and DMSO on CD274 protein levels after LPS treatment for 24 h, representative western blot evaluating cytoplasmic CD274 protein expression from primary human monocytes. (D) Time kinetics of nuclear translocation of NF-κB after LPS treatment. Representative western blot evaluating p65 protein levels in nuclear and cytoplasmic proteins from primary human monocytes. IκB-α protein levels in cytoplasmic proteins indirectly indicates nuclear translocation of NF-κB. LPS was added to cells and incubated for 0, 0.5, 1, 1.5 and 2 h.

To determine the regulation pattern of *CD274* expression in primary human monocytes after LPS treatment, *CD274* at mRNA and protein levels was analyzed by real-time PCR and flow cytometry, respectively. A dramatic, time-dependent increase in *CD274* mRNA levels was observed during the first 12 h after LPS treatment. The *CD274* mRNA level increased between 0 and 2 h and continued to increase to a maximum after 12 h (Fig. 1A). These data suggested that the up-regulation of *CD274* transcription in monocytes was in a time-dependent manner after LPS treatment. There are several key sub-pathway molecules in the LPS-TLR4 classical signal transduction pathway, such as NF-κB, AP-1 and IRF3 [15], but which one may play a more important role is unknown.

NF-κB inhibitor BAY, AP-1 inhibitor T-5224 and PI3K inhibitor Wort were used respectively to assess their effects on individual sub-pathway signal protein activation and *CD274* mRNA accumulation after LPS treatment. Data showed that only NF-κB inhibitor BAY pre-treatment markedly inhibited *CD274* mRNA accumulation in primary human monocytes after LPS treatment for 12 h (Fig. 1B), which indicated that NF-κB may be involved in LPS-induced *CD274* expression, the protein expression assayed by western blotting has consist results with mRNA level data, further corroborating that NF-κB plays a significant role in LPS induced *CD274* gene expression (Fig. 1C). It is known that NF-κB activation mainly occurs via p65/p50 heterodimer nuclear translocation in the LPS-TLR4 signaling pathway. Therefore, we studied the time kinetics of NF-κB activation. Western blotting showed that the cytosol concentrations of p65 and IκB gradually declined during the first 1 h after LPS treatment, then gradually recovered from 1 to 2 h, which corresponded with the trend of nuclear p65 concentration (Fig. 1D). This result indicated that nuclear translocation of p65/p50 heterodimers mainly occurred in the first 1 h after LPS treatment.

**Figure 2:**
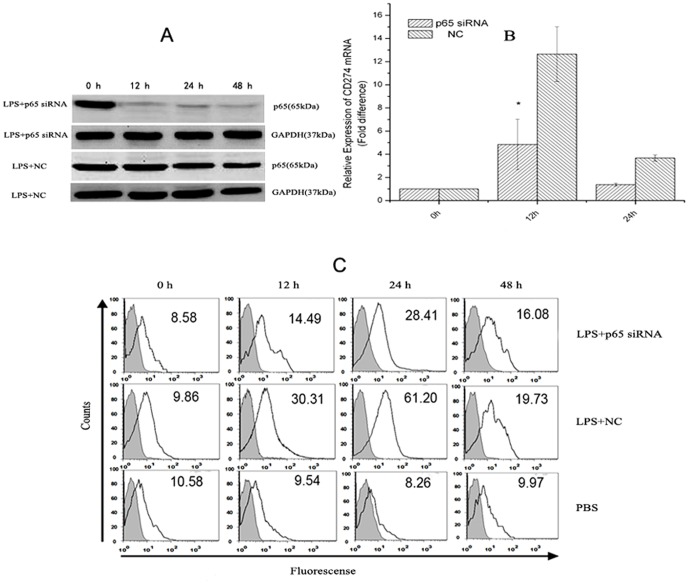
LPS and p65 siRNA combined treatment regulates CD274 at mRNA and protein levels in primary human monocytes. CD274 mRNA levels were determined by quantitative real-time PCR and are shown as fold changes in arbitrary units. (A) Representative western blot evaluating p65 protein levels after siRNA (negative control (NC) or p65-directed) nucleofection of primary human monocytes. LPS was added to cells after 24 h of transfection and incubated for 0, 12, 24 and 48 h. (B) Effect of LPS and p65 siRNA combined treatment on CD274 mRNA levels (n = 3, repeated three times, *P<0.05 vs. control with LPS treatment for 12 h). (C). Effect of LPS and p65 siRNA combined treatment on CD274 protein levels. CD274 protein levels were determined by flow cytometry. Dotted lines indicate background staining. Numbers in histograms indicate the geometric mean fluorescence of CD274-positive monocytes.

To confirm the role of NF-κB in *CD274* transcriptional stimulation by LPS, we silenced endogenous p65 protein expression by a specific siRNA. Primary monocytes were nucleofected with negative control siRNA (NC) or p65 siRNA. An immunoblotting assay showed that p65 was efficiently knocked down after 36 h of siRNA transfection. In addition, p65 siRNA, but not NC siRNA specifically knocked down the expression of p65, but not GAPDH (Fig. 2A). After treatment with p65 siRNA, significantly decreased *CD274* mRNA expression was observed, compared with that of siRNA negative control, following LPS treatment (Fig. 2B). This result was consistent with the inhibitory effect of BAY (Fig. 1B) and demonstrated that p65 knockdown blocked the stimulatory effect of LPS on *CD274* mRNA expression in primary human monocytes. Furthermore, flow cytometric analysis showed that p65 siRNA treatment counteracted the enhancement effect of LPS treatment on CD274 protein levels (Fig. 2C). These findings indicated that the NF-κB signaling sub-pathway played a key role in LPS-induced *CD274* up-regulation in primary human monocytes.

### Identification of the TSS in *CD274*, and Sequence Analysis of the Potential *CD274* Promoter Region

**Figure 3:**
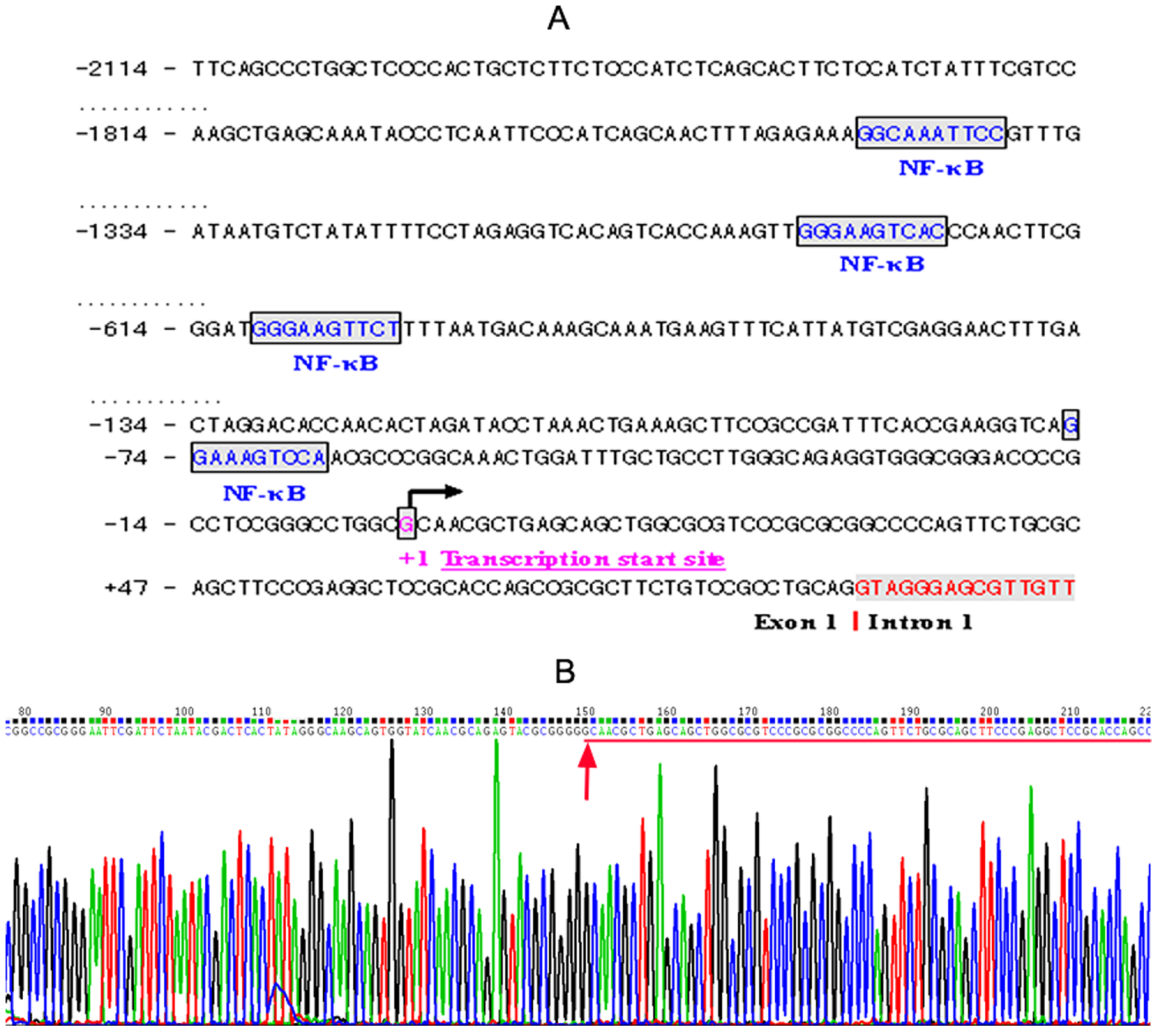
CD274 promoter analysis. (A). In silico analysis of an approximately 2.1 kb nucleotide sequence of the potential promoter and first exon of CD274. The first nucleotide of the 5′-RACE product is indicated by an arrow and assigned the nucleotide position +1. Putative NF-κB binding sites are boxed. (B). Partial sequencing result for the 5′RACE of the CD274 cDNA from a representative clone, red arrow represent the corresponding CD274 transcription start site.

To further study the regulatory mechanism of *CD274* transcription, we needed to identify the exact TSS of *CD274* to locate the genuine promoter region. SMART 5′ RACE was performed using one reverse primer and total RNA isolated from primary human monocytes. Ten clones were produced and sequenced. Comparison of the clone sequences with that of the human genome using BLAST mapped the 5′ end base of the longest clone to Chromosome 9: nucleotide position 5,440,504 (http://www.ensembl.org/Homo_sapiens/Gene/Sequence?g=ENSG00000120217). We assigned this nucleotide in the human genome as the genuine TSS of *CD274* (Fig. 3). The first exon of *CD274* has 91 bp (Ch 9: 5,440,506–5,440,596), intron 1 has 5,503 bp (Ch 9: 5,440,597–5,446,099) and exon 2 has 66 bp (Ch 9: 5,446,100–5,446,165). The sequence of intron 1 complies with the GT-AG rule, and the A of the initiation codon (ATG) is the 15^th^ nucleotide of exon 2 (Ch 9: 5,446,114). This TSS site mapped to 105 nucleotides upstream to the translation start site (ATG) and was located 53 bp upstream of the 5′ end of the longest cDNA sequence in the GenBank database (NM_014143.2).

Polymerase II promoters are generally defined as regions of a few hundred base pairs, which are located directly upstream of the TSS. More distal regions and parts of the 5′ untranslated region may also contain regulatory elements, and may be part of the promoter [16]. The exact length of a promoter can only be defined experimentally. However, for an initial *in silico* analysis it may be sufficient (and also necessary) to restrict the region to approximately 300–1000 bp upstream of the TSS (http://www.genomatix.de/online_help/help_gems/faq.html). Here, we extracted approximately 2100 bp of genomic sequence upstream of the *CD274* TSS. Bioinformatic sequence analysis revealed four putative binding motifs for NF-κB in the *CD274* promoter region (Fig. 3): nt −1,769 to −1,760 (Ch 9: 5,438,737–5,438,746) relative to the TSS, nt −1,293 to −1,284 (Ch 9: 5,439,213–5,439,222), nt −610 to −601 (Ch 9: 5,439,896–5,439,905) and nt −75 to −66 (Ch 9: 5,440,431–5,440,440). A classical TATA or CAAT consensus sequence was not found in this 2.1 kb sequence.

### Identification of Core *CD274* Promoter Activity

**Figure 4:**
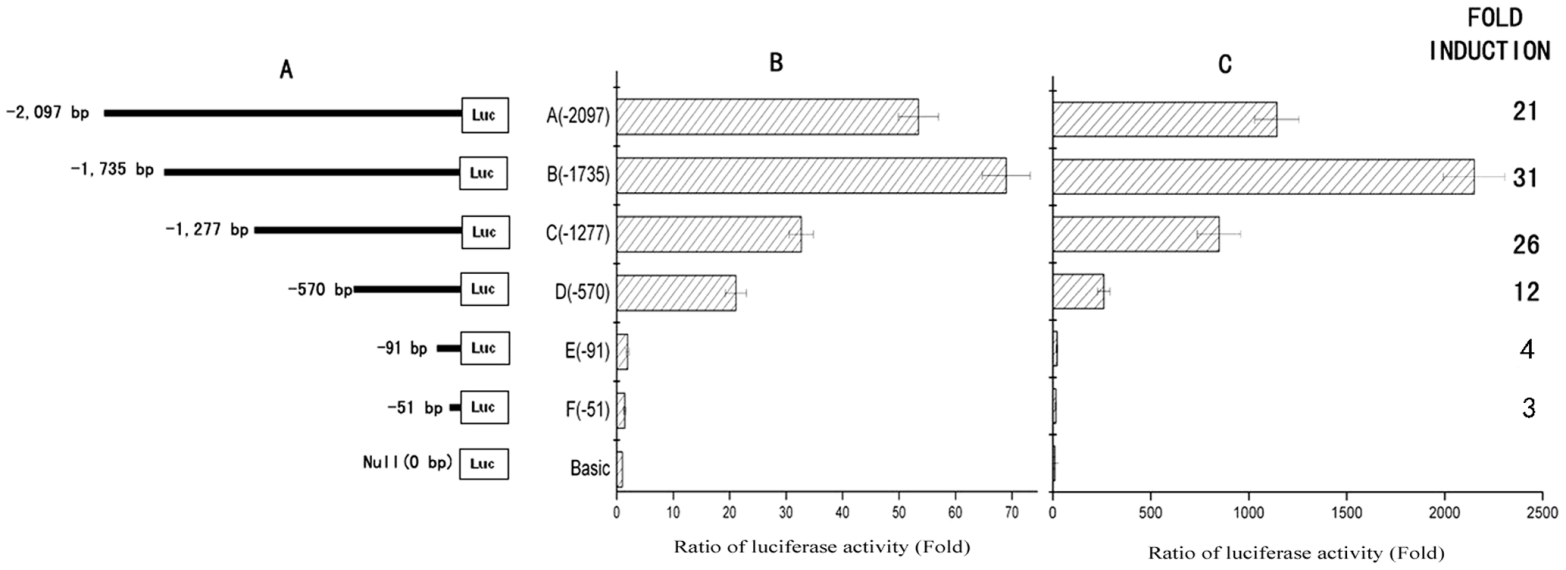
CD274 promoter/reporter deletion analysis. Constructs were generated by progressively cloning 5′-truncated CD274 promoter fragments into the pGL3-basic luciferase vector. Negative numbers denote bp distances from the transcriptional start site (panel A). U937 cells were electroporation cotransfected with these reporter constructs and pRL-TK to control for transfection efficiency. Cells were either unstimulated (panel B) or stimulated with LPS (panel C) for 24 h.

To map the minimal promoter region in the *CD274* gene required for initiation and induction of gene transcription, based on the distribution of potential NF-κB binding sites, luciferase reporter constructs containing progressive deletions of the 2097 bp genomic DNA fragment were generated (Fig. 4). Because NF-κB is significantly activated in LPS-stimulated U937 cells [17], each construct and inner control vector pRL-TK were nucleofected into U937 cells, cultured with or without LPS treatment, and assayed for dual luciferase reporter activity. Our results showed that the 1735 bp fragment induced a large increase in basal luciferase activity and deletions of DNA regions upstream to nt −570 (relative to the TSS) did not significantly decrease basal luciferase expression as compared with that of the full-length 2097 bp fragment (Fig. 4). However, deletion of an additional 479 bp (from nt −570 to −91) reduced reporter activity to a level similar to that obtained with the promoterless luciferase construct (pGL3-Basic) (Fig. 4). These results suggested that the cis-regulatory elements required for basal transcription of the *CD274* gene were located in a 570 bp region upstream of the TSS.

To further identify the role of NF-κB in *CD274* transcription in monocytes upon LPS stimulation, the various deletion constructs were nucleofected into U937 cells and assayed for luciferase activity after LPS treatment. The pGL3-1277Luc construct showed a reporter gene activity similar to that obtained with the full-length pGL3-2097Luc construct. In contrast, cells transfected with pGL3-570Luc, pGL3-91Luc and pGL3-51Luc showed markedly reduced luciferase activity in response to U937 cell activation as compared with those of less deleted constructs (Fig. 4). These data indicated that cis-regulatory elements located between nt −1735 and −570 were required for *CD274* promoter induction, whereas cis-regulatory elements located between nt −570 and −91 were essential for *CD274* basal transcription. Moreover, these results are in good agreement with the distribution of putative binding sites for inducible transcription factors NF-κB present in the promoter region (Fig. 3).

### Determination of a Key Functional NF-κB Site Involved in LPS-induced *CD274* Transcriptional Activity

**Figure 5:**
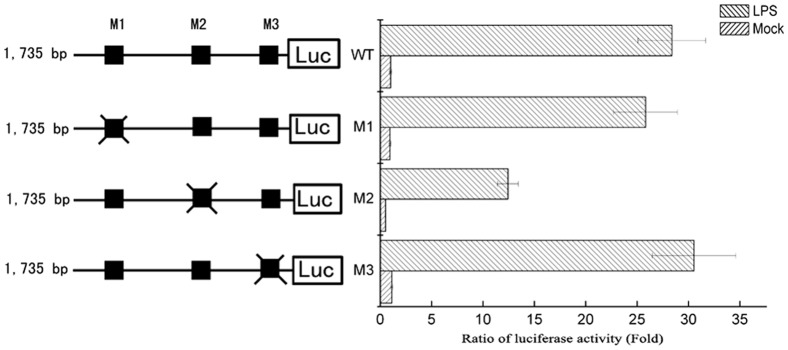
Transcriptional activity of CD274 promoter mutants. Various pGL3-1735Luc mutated constructs were generated. Filled boxes indicate putative NF-κB sites (M1–M3). Mutations in these sites are indicated by hatched boxes. U937 cells were electroporation cotransfected with the indicated reporter construct and pRL-TK to control for transfection efficiency. Cells were treated with or without LPS for 24 h.

To test the functional role of these putative NF-κB binding sites in *CD274* promoter activity, the corresponding residues were independently mutated in the pGL3-1735Luc promoter construct. Mutated reporter constructs and pRL-TK were nucleofected into U937 cells, then basal and LPS-induced luciferase transcriptional activity of the mutated constructs was compared with that of the native pGL3-1735Luc construct. As shown in Fig. 5, mutation of the NF-κB binding site M2, located between nt −610 and −601 (Ch 9: 5,439,896–5,439,905) reduced activation-induced luciferase activity by more than 50% after LPS treatment, whereas M1- and M3-mutated pGL3-1735Luc promoter constructs showed a similar fold induction as compared with that of the wild-type construct. This result suggested that the M2 NF-κB binding site (nt −610 to −601) played a key role in LPS-induced *CD274* transcriptional activity.

**Figure 6:**
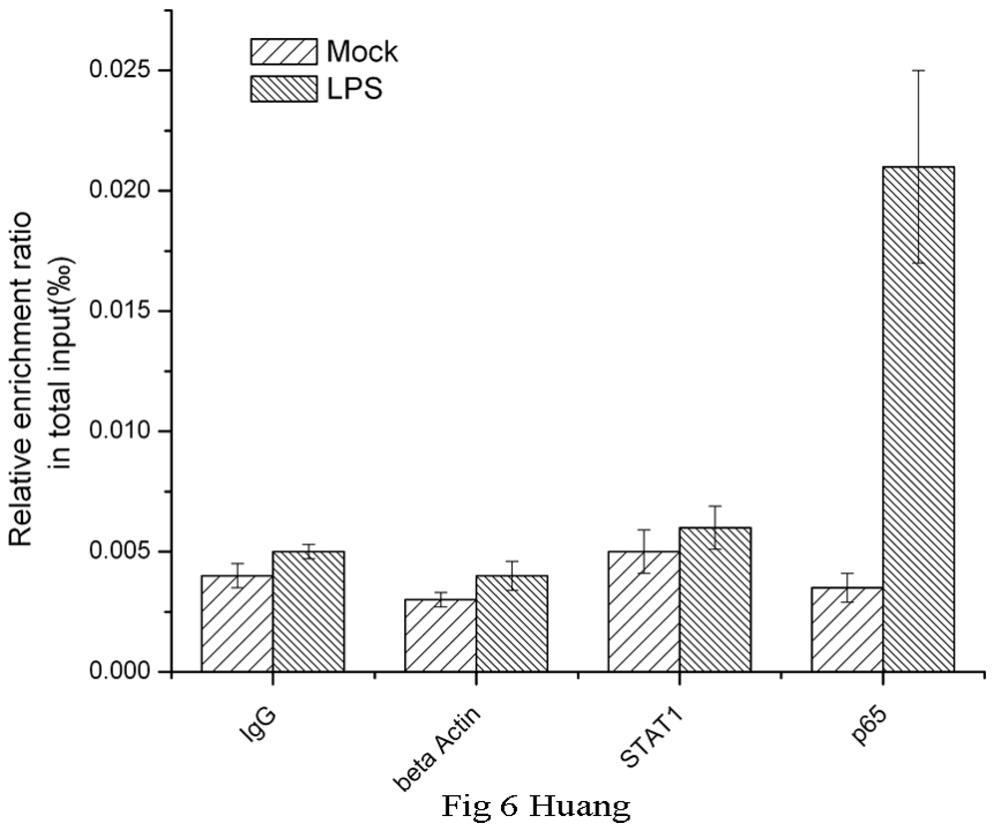
Chromatin immunoprecipitation (ChIP) assays of the CD274 promoter in primary human monocytes. Cells were treated with or without LPS for 1 h. ChIP assays were carried out using an anti-p65 antibody. IgG, anti-β-actin and anti-STAT1 antibodies were used as negative controls. Relative enrichment of each transcription factor-bound DNA was detected by qPCR using ChIP primers. All the results were normalized to input DNA.

To investigate the specific binding of NF-κB to potential NF-κB sites *in vitro,* we used ChIP assays to identify the binding state of NF-κB to these predicted NF-κB binding elements in the *CD274* gene promoter in primary monocytes after LPS treatment. Chromatin from LPS-treated primary monocytes was sonicated and immunoprecipitated with an anti-p65 antibody, and only one fragment containing a possible NF-κB responsive element (nt −610 to −601) was amplified by PCR. As shown in Fig. 6, binding of p65 was specific because immunoprecipitation with anti-β-actin and anti-STAT1 antibodies did not show a detectable *CD274* promoter fragment. This result indicated that NF-κB specifically bound to the M2 NF-κB binding site in the *CD274* promoter and enhanced *CD274* transcription via the p65 component.

These data provided evidence that NF-κB bound to the human *CD274* promoter and regulated transcription in primary human monocytes after LPS treatment, and this regulation was most likely mediated via one of the NF-κB binding sites (nt −610 to −601).

## Discussion

Although our understanding of the pathogenesis of inflammation and sepsis has greatly improved in recent years, the molecular mechanisms that determine the conversion of controllable to uncontrollable inflammatory responses are still largely unknown [18]. Immune dysfunction has been shown to play an important role in the development of uncontrolled inflammatory responses [19]. Recent studies reveal that *CD274*, a co-stimulatory molecule and widely expressed in the MPS, possesses dual functions of co-stimulation of naive T cells and inhibition of activated effector T cells to sustain immune homeostasis [20]. Aberrant expression and dysregulation of *CD274* have been reported during bacterial infection, inflammation and in numerous autoimmune diseases [21], [22], [23]. Importantly, the deregulation of the dual functions of *CD274* appears to be associated with a prolonged and incomplete immune response via attracting naive T cells for activation and impairing the functions of already activated effector T cells [24]. Therefore, development of strategies targeting *CD274* signals provides a novel and promising approach to manipulate the devastating diseases associated with sepsis [7], [20]. Thus, *CD274* may play a critical immunoregulatory role in the control of inflammatory responses.

The MPS plays a central role in the pathogenesis of inflammation and sepsis [25], [26]. However, relatively little is known about the molecular mechanism that controls *CD274* expression in this system. The currently available studies showed that the molecular mechanisms of action regulating the gene expression of *CD274* vary in both different cell types and distinct stimuli. For example, in the process of skin inflammation IFN-γ can induce the expression of *CD274* mRNA in dermal fibroblast cells with NF-κB and MAPK and PI3K signaling pathways involved [27], and LPS-TLR4 interaction induces *CD274* expression through MAPK pathways in bladder cancer cells [28]. Recently, Wolfle SJ and co-workers found that during DC differentiation TLR agonists such as LPS induce a STAT-3-mediated expression of *CD274* and favor the development of tolerogenic APCs [29]. However, during the process of inflammation the detailed molecular mechanism that controls *CD274* expression in MPS is still elusive. It is well known that stimulation of TLR4 induces signaling cascades through MyD88 (myeloid differentiation factor 88) dependent and/or MyD88 independent (TRIF-dependent) pathways, leading eventually to the activation of NF-kB, AP-1, and IRF-3 transcription factors, but which one of the three may play a more important role in inducing the up-regulation of *CD274* expression in MPS is still unknown. If the specific molecular signaling pathway governing the *CD274* gene expression after TLR4 activation in MPS and some of the corresponding promoter sequence of *CD274* gene can be determined, then it might be reasonably helpful to provide novel and significant ways and targets to manipulate inflammatory process via regulating the *CD274* gene expression say using high specific nucleic decoys to block the promoter binding sites.

In the present study, we investigated the relationship between the regulation of *CD274* expression and the TLR4 signaling's sub-pathways in human primary monocytes using LPS as the stimulus. Our data initially demonstrated that induction of *CD274* expression by LPS in human monocytes was NF-κB-dependent. To further explore the underlying molecular mechanism of NF-κB signaling enhancement of *CD274* expression, we focused on NF-κB binding sites located in the *CD274* gene promoter. Our data shown that transcription factor NF-κB may play a key role in inducible *CD274* expression via binding to one of the NF-κB binding sites (nt −610 to −601) in the human *CD274* promoter to regulate transcription in human monocytes after LPS treatment.

In conclusion, the present study suggests that induced *CD274* expression occurs via a key NF-κB motif in its promoter in the MPS, which results in an enhanced *CD274* level to regulate inflammation and immune responses. This study establishes a molecular basis to further understand the mechanisms governing *CD274* expression and may provide a novel insight for development of manipulations that control the signaling cascade resulting in *CD274* production in conditions characterized by immunopathological activation of the MPS such as bacterial infection, inflammation and autoimmune diseases.

## Supporting Information

Figure S1 The results determining the appropriate concentration of p65 siRNA nucleofected in primary human monocytes. (A). Representative western blot evaluating p65 protein levels after a series of concentrations (indicated as (0, 20, 40, 60, 80, 100 nM)) of p65 siRNA and negative control (NC) were nucleofected into primary human monocytes for 48 h. (B). Cell viability in the same series of p65 siRNA and NC concentrations were detected by CCK-8 after they were nucleofected into primary human monocytes for 48 h.
(TIF)

## References

[1] Wang TS, Deng JC (2008) Molecular and cellular aspects of sepsis-induced immunosuppression. J Mol Med 86: 495–506. 18259721 10.1007/s00109-007-0300-4

[2] Castellheim A, Brekke OL, Espevik T, Harboe M, Mollnes TE (2009) Innate immune responses to danger signals in systemic inflammatory response syndrome and sepsis. Scand J Immunol 69: 479–491. 19439008 10.1111/j.1365-3083.2009.02255.x

[3] Lenz A, Franklin GA, Cheadle WG (2007) Systemic inflammation after trauma. Injury 38: 1336–1345. 18048040 10.1016/j.injury.2007.10.003

[4] Chang ZL (2009) Recent development of the mononuclear phagocyte system: in memory of Metchnikoff and Ehrlich on the 100th Anniversary of the 1908 Nobel Prize in Physiology or Medicine. Biol Cell 101: 709–721. 19743965 10.1042/BC20080227

[5] Freeman GJ, Long AJ, Iwai Y, Bourque K, Chernova T, et al (2000) Engagement of the PD-1 immunoinhibitory receptor by a novel B7 family member leads to negative regulation of lymphocyte activation. J Exp Med 192: 1027–1034. 11015443 10.1084/jem.192.7.1027 PMC2193311

[6] Dong H, Chen X (2006) Immunoregulatory role of B7-H1 in chronicity of inflammatory responses. Cell Mol Immunol 3: 179–187. 16893498 PMC2186063

[7] Okazaki T, Honjo T (2007) PD-1 and PD-1 ligands: from discovery to clinical application. Int Immunol 19: 813–824. 17606980 10.1093/intimm/dxm057

[8] Riley JL (2009) PD-1 signaling in primary T cells. Immunol Rev 229: 114–125. 19426218 10.1111/j.1600-065X.2009.00767.x PMC3424066

[9] Yamazaki T, Akiba H, Iwai H, Matsuda H, Aoki M, et al (2002) Expression of programmed death 1 ligands by murine T cells and APC. J Immunol 169: 5538–5545. 12421930 10.4049/jimmunol.169.10.5538

[10] Lien E, Means TK, Heine H, Yoshimura A, Kusumoto S, et al (2000) Toll-like receptor 4 imparts ligand-specific recognition of bacterial lipopolysaccharide. J Clin Invest 105: 497–504. 10683379 10.1172/JCI8541 PMC289161

[11] Kawai T, Akira S (2007) Signaling to NF-kappaB by Toll-like receptors. Trends Mol Med 13: 460–469. 18029230 10.1016/j.molmed.2007.09.002

[12] Akhter A, Gavrilin MA, Frantz L, Washington S, Ditty C, et al (2009) Caspase-7 activation by the Nlrc4/Ipaf inflammasome restricts Legionella pneumophila infection. PLoS Pathog 5: e1000361. 19343209 10.1371/journal.ppat.1000361 PMC2657210

[13] Gavrilin MA, Mitra S, Seshadri S, Nateri J, Berhe F, et al (2009) Pyrin critical to macrophage IL-1beta response to Francisella challenge. J Immunol 182: 7982–7989. 19494323 10.4049/jimmunol.0803073 PMC3964683

[14] Matys V, Fricke E, Geffers R, Gossling E, Haubrock M, et al (2003) TRANSFAC: transcriptional regulation, from patterns to profiles. Nucleic Acids Res 31: 374–378. 12520026 10.1093/nar/gkg108 PMC165555

[15] Lu YC, Yeh WC, Ohashi PS (2008) LPS/TLR4 signal transduction pathway. Cytokine 42: 145–151. 18304834 10.1016/j.cyto.2008.01.006

[16] Rippe K, von Hippel PH, Langowski J (1995) Action at a distance: DNA-looping and initiation of transcription. Trends Biochem Sci 20: 500–506. 8571451 10.1016/s0968-0004(00)89117-3

[17] Legrand-Poels S, Maniglia S, Boelaert JR, Piette J (1997) Activation of the transcription factor NF-kappaB in lipopolysaccharide-stimulated U937 cells. Biochem Pharmacol 53: 339–346. 9065737 10.1016/s0006-2952(96)00715-0

[18] Kumar V, Sharma A (2009) Is neuroimmunomodulation a future therapeutic approach for sepsis? Int Immunopharmacol. 10.1016/j.intimp.2009.10.003 19840870

[19] Biswas SK, Lopez-Collazo E (2009) Endotoxin tolerance: new mechanisms, molecules and clinical significance. Trends Immunol 30: 475–487. 19781994 10.1016/j.it.2009.07.009

[20] Keir ME, Butte MJ, Freeman GJ, Sharpe AH (2008) PD-1 and its ligands in tolerance and immunity. Annu Rev Immunol 26: 677–704. 18173375 10.1146/annurev.immunol.26.021607.090331 PMC10637733

[21] Maier LM, Hafler DA (2009) Autoimmunity risk alleles in costimulation pathways. Immunol Rev 229: 322–336. 19426231 10.1111/j.1600-065X.2009.00777.x

[22] Pentcheva-Hoang T, Corse E, Allison JP (2009) Negative regulators of T-cell activation: potential targets for therapeutic intervention in cancer, autoimmune disease, and persistent infections. Immunol Rev 229: 67–87. 19426215 10.1111/j.1600-065X.2009.00763.x

[23] Podojil JR, Miller SD (2009) Molecular mechanisms of T-cell receptor and costimulatory molecule ligation/blockade in autoimmune disease therapy. Immunol Rev 229: 337–355. 19426232 10.1111/j.1600-065X.2009.00773.x PMC2845642

[24] Seo SK, Jeong HY, Park SG, Lee SW, Choi IW, et al (2008) Blockade of endogenous B7-H1 suppresses antibacterial protection after primary Listeria monocytogenes infection. Immunology 123: 90–99. 17971153 10.1111/j.1365-2567.2007.02708.x PMC2433284

[25] Paukov VS, Daabul SA, Belaieva N (2005) [The role of macrophages in pathogenesis of limited inflammation]. Arkh Patol 67: 3–10. 16209290

[26] Fujiwara N, Kobayashi K (2005) Macrophages in inflammation. Curr Drug Targets Inflamm Allergy 4: 281–286. 16101534 10.2174/1568010054022024

[27] Lee SK, Seo SH, Kim BS, Kim CD, Lee JH, et al (2005) IFN-gamma regulates the expression of B7-H1 in dermal fibroblast cells. J Dermatol Sci 40: 95–103. 16085391 10.1016/j.jdermsci.2005.06.008

[28] Qian Y, Deng J, Geng L, Xie H, Jiang G, et al (2008) TLR4 signaling induces B7-H1 expression through MAPK pathways in bladder cancer cells. Cancer Invest 26: 816–821. 18608206 10.1080/07357900801941852

[29] Wolfle SJ, Strebovsky J, Bartz H, Sahr A, Arnold C, et al (2011) PD-L1 expression on tolerogenic APCs is controlled by STAT-3. Eur J Immunol 41: 413–424. 21268011 10.1002/eji.201040979

