# Supplementary figures and images for: NF-κB Plays a Key Role in Inducing CD274 Expression in Human Monocytes after Lipopolysaccharide Treatment
Source: PLoS One. 2013 Apr 9;8(4):e61602. doi: 10.1371/journal.pone.0061602 (PMC3622012; doi:10.1371/journal.pone.0061602)

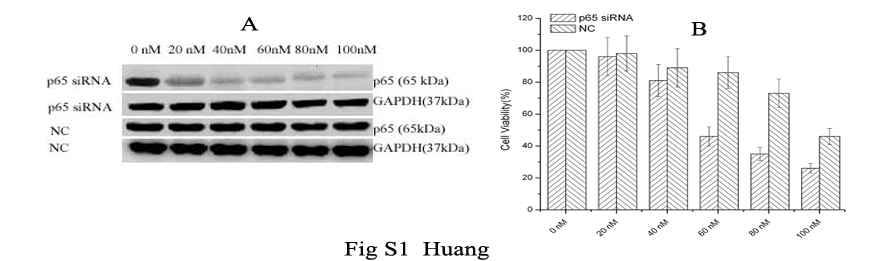

Supplement: Figure S1 — The results determining the appropriate concentration of p65 siRNA nucleofected in primary human monocytes. (A). Representative western blot evaluating p65 protein levels after a series of concentrations (indicated as (0, 20, 40, 60, 80, 100 nM)) of p65 siRNA and negative control (NC) were nucleofected into primary human monocytes for 48 h. (B). Cell viability in the same series of p65 siRNA and NC concentrations were detected by CCK-8 after they were nucleofected into primary human monocytes for 48 h. (TIF) [file pone.0061602.s001.tif]
